# Clinical characteristics and prognosis of Total Rhegmatogenous retinal detachment: a matched case-control study

**DOI:** 10.1186/s12886-020-01560-4

**Published:** 2020-07-13

**Authors:** Jae-Yun Sung, Min-Woo Lee, Yeo-Kyoung Won, Hyung-Bin Lim, Jung-Yeul Kim

**Affiliations:** 1 Department of Ophthalmology, Chungnam National University Sejong Hospital, 20, Bodeum 7-ro, Sejong-si, Republic of Korea; 2 Department of Ophthalmology, Konyang University Hospital, 158, Gwanjeodong-ro, Seo-gu, Daejeon Republic of Korea; 3 Department of Ophthalmology, Chungnam National University Hospital, 282, Munhwa-ro, Jung-gu, Daejeon Republic of Korea

**Keywords:** Total rhegmatogenous retinal detachment, Proliferative vitreoretonotaphy, Ocular trauma, Risk factors

## Abstract

**Background:**

Although many studies have reported clinical features, surgical outcomes of rhegmatogenous retinal detachment (RRD), studies focusing on total RRD are rare**.** In this study, we investigate the clinical characteristics, risk factors, and prognosis of total RRD.

**Methods:**

A retrospective chart review was performed on cases of 44 total RRD and an age- and sex-matched 88 partial RRD. Two groups were compared for clinical characteristics, risk factors, and prognosis.

**Results:**

The prevalence of total RRD in all cases of retinal detachment was 4.4%. Pseudophakic eye, ocular trauma, and proliferative vitreoretinopathy (PVR) were significantly associated with a risk of total RRD (***P*** = .002, ***P*** = .003, and ***P*** < .001, respectively). In the total RRD group, retinal breaks were located in both superior and inferior parts of the retina, and macular holes and giant retinal tears were frequently found. The best-corrected visual acuity (log MAR) before surgery and final best-corrected visual acuity after surgery were 2.23 ± 0.45 and 1.88 ± 0.96, which was significantly poorer than in the partial RRD group (***P*** < .001). The success rate after primary surgery was 75.0% in the total RRD group, which was significantly lower than partial RRD group (***P*** < .001). Old age, pseudophakic eye, and macular hole as the type of retinal break were highly associated with low success rate. (***P*** = .010, ***P*** = .0500, and ***P*** = .002).

**Conclusions:**

Patients with total RRD had higher recurrence rate and poorer visual outcome after surgery than patients with focal RRD. Old age, pseudophakic eye, and presence of macular hole were important risk factors for recurrence after total RRD repair. Additional surgical procedures should be considered to combine with vitrectomy to achieve better surgical outcomes in these patients.

## Background

Rhegmatogenous retinal detachment (RRD) can cause permanent vision loss. Vitreous traction creates a retinal break allowing liquefied vitreous humor to access the subretinal space and separate the pigmented epithelial and sensory retinal layers [1–3]. The prevalence of RRD ranges from 6.3 to 17.9 per 100,000 subjects annually. It is more common in males than females and can develop at any age but is more frequent in those aged 20–60 years old [4–6]. Myopia, lattice degeneration, inflammation, retinal injury, atopic dermatitis, and posterior vitreous detachment are identified as predisposing risk factors in RRD [7]. RRD requires surgical intervention, and the aim of the surgery is to reattach the retina by the break closure and traction relief. Pneumatic retinopexy, scleral buckling, and vitrectomy have been used successfully for the treatment of RRD, with high success rates recently [8–11]. The factors affecting the postoperative success include the extent of retinal detachment, location of retinal tear, lens status, myopia and preoperative visual acuity [12–15].

Many studies have evaluated the clinical features, risk factors and surgical success rates of RRD, but studies focused only on total RRD has not been reported yet. In this retrospective study, we investigated the prevalence, clinical characteristics, risk factors, and prognosis of total RRD compared to partial RRD, and identified preoperative parameters affecting success rate by subgroup analysis.

## Methods

We retrospectively reviewed the medical records of patients who underwent surgery for RRD at the Department of Ophthalmology, Chungnam National University Hospital, South Korea, from January 2007 to June 2016. The study followed all relevant tenets of the Declaration of Helsinki, and the protocol was approved by the institutional review board of the hospital.

Inclusion criteria were patients who underwent surgery for rhegmatogenous retinal detachment and followed-up at least 6 months after surgery. Exclusion criteria were patients who underwent any other ocular surgery except simple cataract surgery and patients with other ocular disease, such as tractional or exudative retinal detachments, macular degeneration, retinal vein occlusion. All patients underwent a detailed ophthalmological history-taking, a preoperative examination including best-corrected visual acuity, intraocular pressure, axial length measurements, slit-lamp and indirect fundus examination. The extent of RRD and the types, locations, and numbers of retinal breaks were recorded. The retina was divided into four quadrants centered at the fovea. Break locations were classified as superior, inferior, and both superior and inferior. If a break was located on the horizontal line that connected the fovea, the break was classified as superior. A refraction > − 6 diopters or an axial length > 26 mm were considered as high myopia.

### Groups of patients

When the retina was detached over all four quadrants, we defined this as total RRD (study group). If the extent of detachment was less, we defined this as partial RRD (control group). Partial RRD group were randomly matched by age- and sex to the total RRD group. We also performed subgroup analyses in total RRD group by success/failure to identify preoperative factors affecting success rate.

### Surgical procedures

Pars plana vitrectomy (PPV) only, or PPV combined with scleral buckling (SB) was performed by two trained operators (JYK, YJJ). We considered to perform PPV with SB in total RRD patients who present with preoperative PVR, trauma, or multiple retinal tears. When scleral buckling was combined, either a sponge or band was used to perform the scleral buckling or encircling, depending on the fundoscopic findings. PPV (23-gauge) was performed using an Accurus Surgical System (Alcon Laboratories, Inc., Fort Worth, TX) until 2013 and a Constellation Vision System (Alcon Laboratories, Inc.) since that time. Cataract operations with IOL implantations were performed with vitrectomy if there were significant cataracts. Perfluorocarbon was injected until its bubble meniscus reached the posterior edge of the retinal break in all eyes using a blunt cannula. Fluid–air exchange was performed while draining subretinal fluid (SRF) through retinal breaks. In eyes with only macular hole, small peripheral retinotomy was performed to drain SRF. SRF was completely drained and PFCL was removed. Endolaser was done around the break after removing PFCL to minimize the risk of subretinal PFCL. Sulfur hexafluoride (SF_6_), perfluoropropane (C_3_F_8_), or silicon oil were used as tamponades. The selection of tamponade agent was based on the characteristics of retinal detachment and expected patient compliance. Complicated cases such as retinal detachment with PVR, giant retinal tear, or macular hole were considered to use silicone oil. Monocular patients and patients who were difficult to maintain facedown position after surgery were also considered to use silicone oil. Although in these complicated cases including macular hole, long-acting gas injection was considered when the patient strongly refused to have second surgery after explaining the need for a second procedure to remove the oil. Patients with macular hole were treated by vitrectomy with internal limiting membrane (ILM) peeling, and either gas or silicone oil tamponade. Primary success was defined as retinal reattachment after a single operation and persisted for > 6 months. Failure was defined as recurred retinal detachment in anywhere.

### Statistical analysis

We transformed Snellen visual acuities into the logarithms of the maximum angle of resolution (log MAR). All analyses were performed using SPSS version 12.0 software (SPSS Inc., Chicago, IL). Student’s *t*-test, the Mann-Whitney U-test, the chi-square test, Fisher’s exact test, and logistic regression analyses were used. A *p*-value < 0.05 was considered to reflect statistical significance.

## Results

### Clinical characteristics

Out of 994 patients, diagnosed with RRD and underwent surgery from January 2007 to June 2016, 44 (4.4%) were diagnosed with total RRD. Among the 44 patients with total RRD, 27 patients were men (61.0%). The mean age was 51.73 years old and the mean follow-up period after surgery was 28.61 months.

Nine eyes (20.5%) had histories of ocular trauma and 16 (36.4%) were found to have Proliferative vitreoretinopathy (PVR) in preoperative examinations. Eighteen eyes were pseudophakic (40.9%) and 12 eyes were high myopia (27.3%). The total RRD group had more histories of ocular injuries than the partial RRD group (***P*** = .003). Of nine patients, eight had eye perforations and one had blunt trauma. Significantly more PVR and pseudophakic eye were found in the total RRD group than in the partial RRD group (***P*** = .014, ***P*** < .001, respectively). The mean symptom duration was 34.06 days (range 1 to 180 days) in the total RRD group, significantly longer than in the partial RRD group (9.03 days) (***P*** < .001) **(**Table 1**).**

**Table 1:** Clinical characteristics of patients in total rhegmatogenous retinal detachment (RRD) group and partial RRD group

	Total RRD (*n* = 44)	Partial RRD (*n* = 88)	*p*-value
Right:Left (n)	20:24	43:45	0.712^a^
Premedical history (n, %)			
Hypertension	11 (25.0%)	25 (28.4%)	0.678^a^
Diabetes mellitus	0 (0.0%)	6 (6.8%)	0.178^b^
Trauma history (n, %)	9 (20.5%)	3 (3.4%)	**0.003**^b^
Proliferative vitreoretinopathy (n, %)	16 (36.4%)	6 (6.8%)	**< 0.00**^b^
Pseudophakic eye (n, %)	18 (40.9%)	14 (15.9%)	**0.002**^a^
High myopia (≥ 26 mm) (n, %)	12 (27.3%)	24 (27.3%)	0.784^a^
Duration of symptom (days, mean ± SD)	34.06 ± 46.87	9.03 ± 11.46	**< 0.001**^c^

*SD* standard deviation

Significant *P* values are expressed in bold characters

^a^chi-square test

^b^fisher’s exact test

^c^independent t test

### Characteristics of retinal breaks

In the total RRD group, 25 eyes had a single break (56.8%) and 19 had two or more breaks (43.2%); more breaks were found in the total RRD group, but the difference was not statistically significant. (43.2% vs. 33.0%, ***P*** = .250). In terms of break location, 25 (56.9%) were in the superior part of the retina, and 7 (15.9%) were in the inferior part. In 12 eyes (27.3%), breaks were found in both the superior and inferior parts of retina. Superior breaks were most common in both groups, and the total RRD group had significantly more breaks which were located in both superior and inferior part of the retina. (***P*** < .001). In the total RRD group, 24 eyes had retinal tears (54.6%), 6 had giant retinal tears, 6 had macular holes (13.6%), and 2 had atrophic holes (4.6%). Six eyes had mixed type (13.6%), among those eyes 3 had tears with atrophic holes, and the other 3 had tears with oradialysis. Three of the six eyes (50%) with macular hole were found to have additional retinal tear. The other 3 eyes with only macular hole and no additional retinal tear were all high myopia with posterior staphyloma.

The type of retinal tear was significantly different between two groups. Macular holes and giant retinal tears tended to be found in the total RRD group, and most atrophic holes were found in the partial RRD group (***P*** < .001) (Table 2). In the partial RRD group, 48 out of 88 eyes (55%) had macular-off retinal detachment.

**Table 2:** Characteristics of retinal breaks in total rhegmatogenous retinal detachment (RRD) group and partial RRD group

	Total RRD	Partial RRD	*p*-value
Number of retinal break (n, %)			0.250^a^
Single	25 (56.8%)	59 (67.1%)	
Multiple	19 (43.2%)	29 (32.9%)	
Location of retinal break (n, %)			**0.009**^**a**^
Superior	25 (56.8%)	57 (64.8%)	
Inferior	7 (15.9%)	24 (27.3%)	
Both superior and inferior	12 (27.3%)	7 (7.95%)	
Type of retinal break (n, %)			**< 0.001**^**b**^
Macular hole	6 (13.6%)	0 (0%)	
Atropic hole	2 (4.5%)	24 (27.3%)	
Retinal tear	24 (54.5%)	61 (69.3%)	
Giant retinal tear	6 (13.6%)	2 (2.3%)	
Mixed type	6 (13.6%)	1 (1.1%)	

Significant *P* values are expressed in bold characters

^a^chi-square test

^b^fisher’s exact test

### Anatomical success

In 44 eyes with total RRD, 33 achieved attachment after primary surgery and the success rate was 75%. The anatomical success rate was 96.6% in the partial RRD group, which was significantly higher than that of total RRD group (***P*** < .001). The mean time to recurrence in the total RRD group was 52 days, which was not different between the two groups (***P*** = .859). The best-corrected visual acuities both before and after surgery were lower in the total RRD group (***P*** < .001) (Table 3).

**Table 3:** Surgical results in total rhegmatogenous retinal detachment (RRD) group and partial RRD group

	Total RRD	Partial RRD	*p*-value
Preoperative BCVA (log MAR)	2.23 ± 0.45	0.82 ± 0.83	**< 0.001**^**a**^
Final BCVA (log MAR)	1.88 ± 0.96	0.35 ± 0.52	**< 0.001**^**a**^
Retinal re-detachment after surgery (n)	11/44	3/88	
Success rate (%)	75.0	96.6	**< 0.001**^**a**^
Re-operation (n)	9/11	3/3	
Mean time of re-detachment after primary surgery (months)	52.00 ± 61.95	45.33 ± 11.68	0.859^a^

*logMAR* logarithm of the minimum angle of resolution; *BCVA* best-corrected visual acuity

Significant *P* values are expressed in bold characters

^a^independent t test

In the total RRD group, 11 patients failed to achieve attachment, and nine underwent second surgery. New retinal breaks were found in four eyes, PVR in one, new macular holes in two, and reopening of prior macular holes in two eyes. In recurred cases, vitrectomy accompanied by silicon oil injection was performed on eight eyes, and gas injection was done on one; retinal reattachment after second surgery was achieved in six eyes (Table 4). The final anatomical success rate was 88.63%. In the partial RRD group, 3 eyes had recurrent detachment, and all underwent second surgery. All patients achieved reattachment after reoperation.

**Table 4:** Characteristics of re-detached patients in total rhegmatogenous retinal detachment group

	Initial surgery			Second surgery			
	Type of surgery	Type of tamponade	Type of retinal break	Type of surgery	Type of retinal break	Type of tamponade	Final status
**1**	PPV	SO	Macular hole	–	–	–	–
**2**	PPV	SO	Macular hole	–	–	–	–
**3**	PPV	C_3_F_8_	Macular hole	PPV	Macular hole	SO	Attached
**4**	PPV	C_3_F_8_	Retinal tear	PPV	Retinal tear	SO	Attached
**5**	PPV	C_3_F_8_	Retinal tear	PPV	Retinal tear	SO	Attached
**6**	PPV	SO	Macular hole	PPV	Macular hole	C_3_F_8_	Detached
**7**	PPV	C_3_F_8_	Macular hole	PPV	Retinal tear	SO	Attached
**8**	PPV	SF_6_	Retinal tear	PPV	Macular hole	SO	Attached
**9**	PPV	SO	Retinal tear PVR	PPV	PVR (C1)	SO	Detached
**10**	PPV with SB	SF_6_	Retinal tear	PPV	Retinal tear	SO	Attached
**11**	PPV	C_3_F_8_	Retinal tear PVR	PPV	Macular hole	SO	Detached

*PPV* pars plana virectomy; *SO* silicone oil; *C*_*3*_*F*_*8*_, Octafluoropropane; *SF*_*6*_ Sulfur hexafluoride; *PVR* Proliferative vitreoretinopathy; *SB* scleral buckle

The total RRD group was divided into success and failure subgroups to identify the preoperative risk factors affecting surgical outcome after RRD repair. The success rates were significantly lower in older patients, patients with pseudophakic eyes and with macular holes (***P*** = .01, ***P*** = .05, ***P*** = .002, respectively). In patients with pseudophakic eyes, the risk of redetachment elevated 3.6-times, and in patients with preoperative macular hole, the risk of redetachment increased 9.4-times (Table 5).

**Table 5:** Analysis of the factors predicting anatomic success in total rhegmatogenous retinal detachment group

	Attatched (*n* = 33)	Redetached (*n* = 11)	*P*-value
Age (yr)	47.82	63.45	**0.013**^**a**^
Lens status (n, %)			**0.005**^**b**^
Phakia	23 (69.7%)	2 (18.2%)	
Pseudophakia	10 (33.3%)	9 (81.8%)	
High myopia (n, %)			0.263^c^
No	25 (75.8%)	6 (54.5%)	
Yes	8 (24.2%)	5 (45.5%)	
PVR (n, %)			1.000^c^
No	21 (63.6%)	7 (63.6%)	
Yes	12 (36.4%)	4 (36.4%)	
Trauma (n, %)			0.412^b^
No	25 (75.8%)	10 (90.9%)	
Yes	8 (24.2%)	1 (0.9%)	
Macular hole (n, %)			**0.002**^**b**^
No	32 (97.0%)	6 (54.5%)	
Yes	1 (3%)	5 (45.5%)	
Number of break (n, %)			0.741^c^
Single	20 (60.6%)	6 (54.5%)	
Multiple	13 (39.4%)	5 (45.5%)	
Break location (n, %)			0.343^b^
Superior	18 (54.5%)	8 (72.7%)	
Inferior	7 (21.2%)	0 (0.0%)	
Both superior and inferior	8 (24.2%)	3 (27.3%)	
Type of surgery (n, %)			0.981^b^
Vitrectomy only	28 (84.8%)	10 (90.9%)	
Vitrectomy with SB	5 (15.2%)	1 (9.1%)	
Tamponade (n, %)			
Gas (SF_6_, C_3_F_8_)	16 (48.5%)	7 (63.6%)	0.381^c^
Silicone oil	17 (51.5%)	4 (36.4%)	

*C*_*3*_*F*_*8*_ Perfluoropropane; *SF*_*6*_ Sulfur hexafluoride; *PVR* Proliferative vitreoretinopathy; *SB* scleral buckling

Significant *P* values are expressed in bold characters

^a^independent t test

^b^fisher’s exact test

^c^chi-square test

## Discussion

There have been many studies on RRD but not only on total RRD. Therefore, we analyzed the clinical characteristics, risk factors, and the prognosis in total RRD. RRD usually occurs in patients aged 20 to 80 years old [16, 17], and more common in males [4]. In this study, total RRD was most common in those aged 50–70 years old (mean age 51.73 years) and males were more affected, similar to previous studies.

Patients with pseudophakic eye and history of ocular trauma were at significantly greater risk for total RRD than partial RRD. Cataract surgery can cause posterior vitreous detachment, and blunt trauma can impose traction on the vitreous, increasing the risk for RRD [18]. In patients with both risk factors, initial partial RRD may progress to total detachment. Therefore, rapid diagnosis and treatment are essential. The frequency of PVR was significantly higher in the total RRD group. Cardillo et al. [19] found that ocular injuries and Tseng et al. [20] found that RRD > 3 months in duration and retinal detachment in at least three quadrants, were associated with PVR. In this study, the frequency of trauma history was high in the total RRD group, and symptom duration was significantly longer in total RRD group compared with partial RRD group. Thus, because the development of PVR may be affected by multiple factors, it is hard to consider PVR as an independent risk factor for total RRD.

Most studies on RRD have found that superior breaks are more common [21]; we found this to be the case in both groups. Additionally, we found that the total RRD group had more mixed type breaks and more breaks which were located in both superior and inferior part of the retina. Therefore, if only a single break was found during fundus examination in total RRD patients, careful search for any additional breaks would be needed.

The best-corrected visual acuities before and after surgery were significantly lower in the total RRD group. Among the total RRD patients of the present study, only 4 patients (9%) attained visual acuities ≥10/20 after surgery. Jung and Lee [22] analyzed surgical success rates by the extent of retinal detachment. They reported success rates of 100%, when the extent of retinal detachment was one quadrant, falling to 54.8% when three or four quadrants were involved. In this study, the success rate of primary surgery in the total RRD group was 75.0%, which was significantly lower than the partial RRD group (96.6%). The extent of retinal detachment might be considered as one of the factors associated with duration of detachment [23]. In longstanding RRDs, because the detached retina is rigid, shortened, and can form subretinal membrane, it is difficult to obtain anatomic reattachment after surgery [24]. In our study, 45% of recurrences occurred within 1 month after surgery and 91% occurred within 2 months. Richarson et al. [25] evaluated 171 RRD patients who underwent vitrectomy and reported that 71% of patients recurred within 4 weeks and 83% within 6 weeks, and the mean time from surgery to recurrence was 5.3 weeks. Therefore, frequent and thorough fundus examination is needed especially for 2 months after surgery.

To define the factors affecting surgical outcome, we divided the total RRD group into two subgroups: a success and failure group after primary surgery. Patients with pseudophakic eye and macular hole were significantly associated with low success rate. When total RRD was accompanied by macular holes, the success rate was only 16.7%. Ripanedelli et al. [26] reported that posterior episcleral buckle procedure, located in the macular area, resulted in a better anatomical outcome compared with vitrectomy for retinal detachment with macular hole in high myopic eyes with posterior staphyloma. Ryan et al. [27] found that when repairing macular holes, simultaneous removal of the internal limiting membrane increased the closure rate. In addition, many studies have reported that using C_3_F_8_ or silicon oil (not SF_6_) as an intraocular tamponade when treating retinal detachment accompanied by a macular hole afforded better results [28, 29]. Therefore, if a total RRD patient has a macular hole, combined vitrectomy with macular buckle (especially in myopic eyes with posterior staphyloma), removal of the internal limiting membrane, and silicone oil injection may increase the success rate. PVR is one of the known risk factor for failure after RRD repair [23]. Conversely in our study, PVR was not associated with recurrence. As we mentioned at the methods section, we considered to perform PPV with SB in patients with preoperative PVR, so direct comparison whether the presence of PVR affects the surgical outcome would be limited. In our study, the reattachment rate was higher in patients who received PPV with SB than those who received PPV alone in PVR, but it was difficult to find statistical significance because of the small sample size. Further studies with large number of cases are needed to determine whether performing PPV with SB could increase the anatomical success rate in total RRD with PVR.

The limitations of our study include the fact that it was retrospective study and had a small sample size. In addition, all surgeries were performed by two operators using two vitrectomy instruments. However, to the best of our knowledge, this is the first study specifically focusing on total RRD.

## Conclusion

When treating a patient with total RRD, the high recurrence rate and poor visual outcome should be explained before surgery and careful efforts to find additional breaks are needed. Although a single break was found on preoperative fundus examination in total RRD patients, surgeons should consider the high possibility of presence of additional breaks during surgery. Pseudophakic eye and macular hole were important risk factor for poor surgical outcome after total RRD repair. Therefore, additional surgical procedures should be considered to combine with vitrectomy to achieve better surgical outcome in total RRD patients with pseudophakic eye or macular hole.

## Data Availability

The data of the current study are available from the corresponding author on reasonable request.

## Abbreviations

RRD: Rhegmatogenous retinal detachment
PPV: Pars plana vitrectomy
SF_6_: Sulfur hexafluoride
C_3_F_8_: Perfluoropropane
LogMAR: Logarithms of the maximum angle of resolution
PVR: Proliferative vitreoretinopathy
